# Patient-Level Comparison of Surgical TOGS Components and DUCA-Based Textbook Outcome After Open D2 Total Gastrectomy

**DOI:** 10.3390/medicina62101854

**Published:** 2026-09-24

**Authors:** Uğur Topal, Burak Aydoğan, Yunus Kaycı, Mevlüt Harun Ağca, İshak Aydın, Serdar Gümüş, Ahmet Gökhan Sarıtaş, Orçun Yalav, Cem Kaan Parsak

**Affiliations:** 1 Department of Surgical Oncology, Faculty of Medicine, Çukurova University, 01330 Adana, Turkey; seredargumus@hotmail.com (S.G.); cparsak@yahoo.com (C.K.P.); 2 Department of General Surgery, Islahiye State Hospital, 27800 Gaziantep, Turkey; drburakaydogan@gmail.com; 3 Department of Gastroenterological Surgery, Adana City Training and Research Hospital, University of Health Sciences, 01370 Adana, Turkey; ykayci@hotmail.com; 4 Department of General Surgery, Faculty of Medicine, Çukurova University, 01330 Adana, Turkey; dr.mhagcaa@hotmail.com (M.H.A.); drishakaydin@gmail.com (İ.A.); drags0001@hotmail.com (A.G.S.); drorcun2@yahoo.com (O.Y.)

**Keywords:** textbook outcome, gastric cancer, total gastrectomy, TOGS, DUCA-TO

## Abstract

*Background and Objectives*: Textbook outcome (TO) is increasingly used as a composite quality metric in gastric cancer surgery, but different TO frameworks can classify the same patient differently. This study compared surgical and perioperative components of the Textbook Outcome in Gastric Surgery (TOGS) framework with the Original Dutch Upper Gastrointestinal Cancer Audit textbook outcome (Original DUCA-TO) and a Modified DUCA-TO definition using Clavien–Dindo (CD) grade ≥III as the morbidity threshold, focusing on patient-level agreement and discordance. *Methods*: Patients undergoing curative-intent open D2 total gastrectomy for gastric adenocarcinoma at a single tertiary center (January 2015–January 2025) were retrospectively analyzed. TOGS was evaluated using the surgical and perioperative components of the TOGS framework, excluding the multimodality-treatment domain. Original DUCA-TO used CD grade ≥II morbidity as a failure criterion; Modified DUCA-TO used CD grade ≥III, with other components unchanged. Achievement rates, concordance, factors associated with TO achievement, and overall survival were assessed. *Results*: Among 133 patients, TOGS, Original DUCA-TO, and Modified DUCA-TO were achieved in 45.9%, 27.8%, and 52.6%, respectively. Agreement with TOGS improved after raising the DUCA morbidity threshold, with Cohen’s kappa increasing from 0.563 to 0.686; this reclassified 24.8% of patients to achievement and reversed the discordance direction. Higher preoperative hemoglobin was independently associated with greater odds of TOGS and Original DUCA-TO achievement, with adjusted odds ratios (ORs) of 1.50 and 1.67, respectively. In separate exploratory adjusted models, each 50 mL increase in intraoperative blood loss was associated with lower odds of TOGS and Original DUCA-TO achievement (adjusted ORs, 0.13 and 0.11, respectively), as was each 1-unit increase in intraoperative erythrocyte transfusion (adjusted ORs, 0.14 and 0.13, respectively); postoperative erythrocyte transfusion was also associated with lower odds of TOGS achievement (adjusted OR, 0.26). TOGS and Modified DUCA-TO were associated with longer unadjusted survival, but no definition remained independently associated with overall survival after adjustment. *Conclusions*: The evaluated surgical/perioperative TOGS components and DUCA-based TO definitions are related but not interchangeable after open D2 total gastrectomy for gastric adenocarcinoma. The DUCA morbidity threshold substantially affects achievement rates, the direction of discordance, and the interpretation of associated factors, particularly the association with preoperative hemoglobin under a morbidity criterion that includes transfusion-related CD grade II events. Clear reporting of the TO framework, threshold, and failure patterns is essential for cross-cohort comparisons.

## 1. Introduction

Gastric cancer remains one of the leading causes of cancer incidence and mortality worldwide [1]. In non-metastatic disease, curative gastrectomy with D2 lymphadenectomy is the main surgical component of multimodal treatment [2,3]. Total gastrectomy is required for proximal tumors or when an adequate proximal margin cannot be achieved with subtotal resection [3]. Perioperative outcomes after gastrectomy vary according to institutional surgical volume and geographic setting [4,5]. Higher hospital and surgeon volumes have been associated with lower postoperative mortality and, in several studies, lower postoperative morbidity [4]. Moreover, large East Asian series have generally reported lower postoperative mortality and severe morbidity than many Western cohorts [4,5].

Surgical quality assessment has traditionally relied on individual endpoints, including mortality, complications, length of hospital stay, and readmission [6]. However, low-frequency outcomes, such as mortality, may have limited reliability and ranking stability when used alone for interinstitutional comparisons [7]. Accordingly, composite quality metrics, such as textbook outcome (TO), have been developed to capture the ideal postoperative course more comprehensively. The TO concept was first adapted as a composite quality measure in colorectal cancer surgery and later extended to esophagogastric cancer surgery [6,8,9]. In gastric cancer surgery, TO achievement has been associated with longer-term survival in population-based studies [10,11]. In an Ontario population-based analysis, TO achievement was associated with higher 3-year survival and a lower adjusted hazard of death [10]. In a Spanish population-based study, the TO rate was 41.1%, and TO achievement was associated with longer survival [11].

The Dutch Upper Gastrointestinal Cancer Audit textbook outcome (DUCA-TO) framework was introduced in esophagogastric cancer surgery using the Dutch Upper Gastrointestinal Cancer Audit dataset and was subsequently evaluated as a quality-of-care measure in this setting [6,9]. This framework defines TO using ten components, including potentially curative/radical resection at the end of surgery, tumor-negative margins, adequate lymphadenectomy, absence of postoperative morbidity above the selected Clavien–Dindo (CD) threshold, no reintervention, no readmission to the intensive care unit (ICU), acceptable length of hospital stay, no hospital readmission, and no early mortality [6,9]. In the original DUCA-based definition, adequate lymphadenectomy was defined as examination of ≥15 lymph nodes, and postoperative morbidity was defined using a CD grade ≥II threshold [6]. CD grade II includes pharmacological treatment, postoperative blood transfusion, and total parenteral nutrition [12]. Accordingly, the original DUCA morbidity criterion incorporates treatment-requiring grade II events. In contrast, several gastrectomy-specific TO studies have used CD grade ≥III or ≥IIIa thresholds to represent major postoperative morbidity [13,14,15].

More recently, Marrelli et al. proposed the Textbook Outcome in Gastric Surgery (TOGS) index as a gastric-specific composite quality metric [16]. TOGS includes surgical and perioperative domains, such as tumor-negative margins, adequate lymphadenectomy with procedure-specific lymph-node thresholds, absence of major intraoperative complications, no postoperative reintervention, no unplanned ICU admission, no hospital readmission related to a postoperative surgical complication, no mortality within the defined postoperative window, and an oncological/multimodality-treatment domain [16]. DUCA-TO uses a universal threshold of ≥15 examined lymph nodes, whereas TOGS applies procedure-specific thresholds, including ≥25 lymph nodes for total gastrectomy [6,16]. TOGS also differs from DUCA-TO by using 90-day rather than 30-day windows for readmission and mortality [6,16]. In an external validation study from a high-volume Korean center, TOGS achievement was reported in 74.7% of patients and was associated with higher 5-year overall survival [17].

Heterogeneity in gastrectomy TO definitions has been increasingly emphasized. In a scoping review of 45 studies published between 2017 and 2025, Salem et al. reported a median post-gastrectomy TO rate of 58.6% and identified 26 different TO components across the studies [18]. Inadequate lymphadenectomy and CD grade ≥II complications were among the most frequent barriers to TO achievement [18]. Similarly, Carbonell-Morote et al. reported wide variation in TO achievement rates and highlighted the need for greater standardization in gastric cancer surgery TO research [19]. Recent studies have shifted gastric cancer quality assessment from DUCA-based TO toward the gastric-specific TOGS framework, first proposed in the multicenter Italian Gastric Cancer Research Group (GIRCG) cohort by Marrelli et al. and subsequently externally validated by Carbone et al. in a large high-volume Korean cohort [16,17]. Nevertheless, gastrectomy TO definitions remain heterogeneous, and patient-level agreement, direction of discordance, component-level failure patterns, and the classification impact of morbidity-threshold selection remain insufficiently clarified [16,17,18,19].

This study primarily aimed to compare the achievement rates of surgical and perioperative TOGS components and DUCA-based TO definitions at the patient level in a homogeneous cohort undergoing open D2 total gastrectomy for gastric adenocarcinoma. Secondary aims were to evaluate agreement, direction of discordance, component-level failure patterns, factors associated with TO achievement, the impact of changing the DUCA morbidity threshold, and the exploratory association between TO definitions and overall survival.

## 2. Materials and Methods

### 2.1. Study Design and Patient Selection

This retrospective single-center cohort study was conducted at Çukurova University Faculty of Medicine. Institutional surgical records from January 2015 to January 2025 identified 293 radical gastrectomies, comprising 220 total, 59 subtotal, and 14 proximal gastrectomies. Ethics committee approval was obtained for the retrospective analysis of fully de-identified institutional records (Çukurova University Faculty of Medicine Ethics Committee, decision no. 52, 13 March 2026). The study was conducted in accordance with the Declaration of Helsinki, and informed consent was waived owing to the retrospective design.

Patients were eligible if they underwent curative-intent open D2 total gastrectomy for histologically confirmed gastric adenocarcinoma. Curative intent was determined from the operative plan; final pathological margin involvement did not exclude patients and was retained as a TO failure component. Patients were excluded if they underwent subtotal or proximal gastrectomy, had non-adenocarcinoma histology, underwent D1/D1+ lymphadenectomy or non-D2 dissection, or had intraoperatively or pathologically confirmed distant or peritoneal metastasis. The analytic cohort was restricted to patients undergoing open D2 total gastrectomy to maintain procedural homogeneity. This restriction also ensured consistent application of the procedure-specific TOGS lymph-node criterion [16].

Among the 220 patients who underwent total gastrectomy for gastric adenocarcinoma, 5 were excluded because D2 lymphadenectomy had not been performed. Of the remaining 215 patients, 82 were excluded because historical clinical, operative, pathological, and postoperative records were insufficient for assessment of the study eligibility criteria and/or TO components. The final analytic cohort therefore comprised 133 patients who underwent open D2 total gastrectomy for gastric adenocarcinoma. Lymphadenectomy extent was determined primarily from operative records, and pathology reports were used to verify lymph-node yield. Neoadjuvant chemotherapy status was recorded as a binary variable from medical oncology records.

### 2.2. Data Collection and Definitions

Demographic, clinical, laboratory, operative, pathological, and postoperative outcome data were extracted from institutional medical records. Estimated intraoperative blood loss was recorded in milliliters. Intraoperative erythrocyte transfusion was recorded as the number of units administered during surgery, whereas postoperative erythrocyte transfusion requirement was recorded as a binary variable. Preoperative variables included age, sex, body mass index, American Society of Anesthesiologists (ASA) physical status, Charlson Comorbidity Index, neoadjuvant chemotherapy status, hemoglobin, and albumin.

Pathological variables included Lauren classification, tumor grade, tumor size, pathological T and N categories, linitis plastica, lymph-node yield, metastatic lymph-node count, and resection margin status. Pathological T and N categories were reported as pT/pN after upfront surgery and ypT/ypN after neoadjuvant chemotherapy; therefore, p/ypT and p/ypN denote the applicable pathological stages according to treatment sequence. Pretreatment clinical T and N categories were retrieved from preoperative staging records when available. Because standardized pretreatment staging data were not uniformly available across the study period, these variables were summarized descriptively among patients with retrievable data and were not included in the multivariable models.

High-grade tumors were defined as G3–G4. Preoperative anemia was defined as hemoglobin <13.0 g/dL in men and <12.0 g/dL in women. Resection margin status was obtained from final pathology reports and categorized as R0 or R1; no R2 resections were identified. Potentially curative/radical resection at the end of surgery was determined from operative records.

Readmission and ICU-related criteria were assessed in accordance with the published definitions of the respective TO frameworks [6,16]. Emergency department visits without subsequent hospitalization were not considered readmissions. Reintervention was defined as any surgical, endoscopic, or radiological intervention for a postoperative complication within 30 days after surgery.

Tumor size was summarized among patients with measurable discrete tumors on final pathology, and linitis plastica was recorded as a separate pathological variable. Variables required for evaluating surgical/perioperative TO definitions and the primary multivariable models were complete. Variables used only in exploratory or supplementary analyses were analyzed according to available data, and missingness, when present, was reported in the corresponding Supplementary Tables S1–S5.

### 2.3. Textbook Outcome Definitions

Three TO definitions were evaluated: TOGS, Original DUCA-TO, and Modified DUCA-TO. These definitions assessed textbook surgical outcomes, focusing on resection quality, lymphadenectomy adequacy, perioperative morbidity, early mortality, readmission, and healthcare utilization. Textbook oncological outcomes, which additionally require standardized assessment of multimodality treatment delivery, such as adjuvant chemotherapy completion and time to chemotherapy, were not evaluated.

TOGS was evaluated using the applicable surgical and perioperative components of the GIRCG TOGS framework [16]. Throughout the comparative analyses, TOGS refers specifically to these evaluated surgical and perioperative components and does not represent the complete TOGS framework, as the multimodality-treatment domain was not assessed. Achievement according to the evaluated surgical/perioperative TOGS components required fulfillment of all of the following criteria:

- R0 resection with a tumor-free margin of ≥1 mm;
- Adequate lymphadenectomy, defined as ≥25 examined lymph nodes for total gastrectomy;
- Absence of intraoperative complications as defined by the TOGS framework;
- No reintervention within 30 days after surgery;
- No unplanned ICU/medium-care unit admission for a life-threatening complication within 30 days after surgery;
- No hospital readmission related to a complication after the surgical procedure within 90 days after surgery;
- No in-hospital and/or 90-day mortality.

Intraoperative complications were defined according to the TOGS framework as complete transection or major injury of major vessels, including the hepatic artery, splenic artery, portal vein, or vena cava, requiring reconstruction; with or without acute major bleeding requiring urgent transfusion; or complete transection or major injury of organs, including the bile duct, requiring reconstruction or resection [16]. CD grade was not included as a direct TOGS component. The multimodality-treatment domain of the full TOGS framework includes tumor board evaluation and, when indicated in locally advanced disease, staging laparoscopy and/or compliance with neoadjuvant or perioperative chemotherapy according to the guidelines [16]. This domain was not evaluated because the present study focused on textbook surgical outcome rather than textbook oncological outcome. Original DUCA-TO was defined using ten components [6]. Original DUCA-TO achievement required fulfillment of all of the following components:

- Potentially curative/radical resection at the end of surgery;
- Absence of intraoperative complications;
- Tumor-negative resection margins;
- Examination of ≥15 lymph nodes;
- Absence of CD grade ≥II postoperative complications;
- No reintervention within 30 days after surgery;
- No readmission to the ICU within 30 days after surgery;
- Hospital stay ≤21 days;
- No postoperative mortality within 30 days after surgery;
- No hospital readmission within 30 days after discharge.

Modified DUCA-TO was defined identically to Original DUCA-TO, except that the morbidity threshold was changed from CD grade ≥II to ≥III. The CD grade ≥III threshold was selected for this sensitivity analysis because it distinguishes major complications requiring surgical, endoscopic, or radiological intervention from grade II events, which include pharmacological treatment and blood transfusion [12]. This modification was designed to assess the classification impact of excluding treatment-requiring grade II events, including transfusion, while keeping all other DUCA-TO components unchanged. In addition, CD grade ≥III or ≥IIIa thresholds have been used as major-morbidity components in several gastrectomy-specific textbook outcome studies [13,14,15]. The modified definition was therefore intended to evaluate the sensitivity of DUCA-TO classification to morbidity-threshold selection rather than to propose a replacement for the original DUCA framework. TO was coded as achieved only when all components were fulfilled; failure of any component classified the patient as a non-achiever. Component failures were not mutually exclusive.

### 2.4. Survival Outcome

Overall survival was defined as the interval from surgery to death from any cause or last follow-up. Patients alive at the last follow-up were censored on the date they were last known to be alive. The final vital-status assessment date was 1 January 2026. Mortality status was determined from institutional electronic medical records; linkage to a national mortality registry was not available. Median follow-up was calculated using the reverse Kaplan–Meier method.

### 2.5. Statistical Analysis

Continuous variables were summarized as median [interquartile range (IQR)] and compared using the Mann–Whitney U test because of skewed distributions and limited sample size. Categorical variables were summarized as *n* (%) and compared using the Pearson chi-square or Fisher’s exact test, as appropriate.

Achievement rates, component-level failures, and single-failure mechanisms were calculated separately for TOGS, Original DUCA-TO, and Modified DUCA-TO. Because component failures were not mutually exclusive, component-level percentages could exceed the overall non-achievement rate. Patient-level agreement between definitions was assessed using concordance rates, discordance rates, Cohen’s kappa, and McNemar tests.

Univariable logistic regression was used to evaluate selected clinical, pathological, laboratory, and operative factors associated with TO achievement. Primary multivariable logistic regression models were restricted to prespecified clinically available perioperative variables to avoid overfitting, given the limited number of Original DUCA-TO achievements. Pathological tumor-burden variables were evaluated in univariable analyses and survival adjustment but were not included in the primary logistic models. Multivariable logistic regression models included age, ASA physical status ≥III, preoperative hemoglobin, and neoadjuvant chemotherapy. In additional exploratory analyses, intraoperative blood loss, intraoperative erythrocyte transfusion, and postoperative erythrocyte transfusion requirement were first evaluated using univariable logistic regression. Each variable was then evaluated separately in an additional multivariable model for TOGS and Original DUCA-TO achievement, adjusted for age, ASA physical status ≥III, preoperative hemoglobin, and neoadjuvant chemotherapy. Intraoperative blood loss was modeled per 50 mL increase, intraoperative erythrocyte transfusion per 1-unit increase, and postoperative erythrocyte transfusion requirement as a binary variable. The association between intraoperative blood loss and intraoperative erythrocyte transfusion was assessed using Spearman rank correlation; because of their strong correlation, these variables were not entered simultaneously into the same adjusted model. When complete separation precluded reliable maximum-likelihood estimation, the corresponding adjusted association was reported as not estimable. Odds ratios (ORs) with 95% confidence intervals (CIs) were reported.

Receiver operating characteristic (ROC) analysis was used to evaluate the discriminative performance of preoperative hemoglobin for predicting TOGS achievement. The optimal cut-off was determined using the Youden index, and the 95% confidence interval for the area under the curve (AUC) was estimated using a 2000-resample non-parametric bootstrap.

Overall survival was estimated using the Kaplan–Meier method, and survival curves were compared using the log-rank test. Cox proportional hazards regression was used in exploratory analyses to evaluate the association between TO achievement and overall survival after adjustment for ASA physical status ≥III, p/ypT ≥3 disease, preoperative hemoglobin, and neoadjuvant chemotherapy. Hazard ratios (HRs) with 95% CIs were reported. The proportional hazards assumption was assessed using Schoenfeld residuals. Analyses were performed using Python 3.12 with the pandas, scipy, statsmodels, scikit-learn, and lifelines libraries. All tests were two-sided, and *p* < 0.05 was considered statistically significant.

## 3. Results

### 3.1. Patient Selection and Baseline Characteristics

During the study period, 293 radical gastrectomies were identified, comprising 220 total, 59 subtotal, and 14 proximal gastrectomies. Among the 59 subtotal gastrectomies, 23 were performed for gastrointestinal stromal tumor (GIST) and 36 for gastric adenocarcinoma; all 14 proximal gastrectomies were performed for gastric adenocarcinoma. Among the 220 patients who underwent total gastrectomy for gastric adenocarcinoma, 5 were excluded because D2 lymphadenectomy had not been performed. Of the remaining 215 patients, 82 were excluded because historical records were insufficient for assessment of the study eligibility criteria and/or TO components. The final analytic cohort comprised 133 patients who underwent open D2 total gastrectomy for gastric adenocarcinoma. The patient selection flow is shown in Figure 1.

A total of 133 patients who underwent open D2 total gastrectomy for gastric adenocarcinoma were included in the final analysis. The median age was 64 years [IQR, 56–71], and 85 patients (63.9%) were male. ASA physical status ≥III was present in 33 patients (24.8%).

Neoadjuvant chemotherapy had been administered to 60 patients (45.1%). Pretreatment clinical T and N categories were retrievable for 85 of 133 patients (63.9%). Among these patients, 77 (90.6%) had cT3–4 disease and 71 (83.5%) had cN-positive disease. In the overall cohort, p/ypT ≥3 disease was present in 115 patients (86.5%), and p/ypN-positive disease was present in 93 patients (69.9%). The median preoperative hemoglobin and albumin levels were 12.0 g/dL [IQR, 10.3–12.5] and 34.0 g/L [IQR, 28.0–37.7], respectively (Table 1).

Selected non-component clinical, pathological, laboratory, and operative characteristics stratified by TOGS and Original DUCA-TO achievement are provided in Table S1 and Supplementary Table S2.

### 3.2. Achievement Rates and Component-Level Failures of the Evaluated Surgical/Perioperative TOGS Components, Original DUCA-TO, and Modified DUCA-TO

Using the evaluated surgical/perioperative TOGS components, TOGS achievement was observed in 61 patients (45.9%). When the Original DUCA-TO definition was applied, the achievement rate decreased to 37 patients (27.8%). After changing the DUCA-TO morbidity threshold from CD grade ≥II to grade ≥III, Modified DUCA-TO was achieved in 70 patients (52.6%).

The most frequent component-level failure for TOGS was inadequate lymph-node retrieval according to the total gastrectomy-specific threshold of <25 examined lymph nodes. In contrast, the most frequent component-level failure for Original DUCA-TO was CD grade ≥II morbidity.

Changing the morbidity threshold from CD grade ≥II to grade ≥III reduced morbidity-driven failures by 50 patients, from 87 patients (65.4%) to 37 patients (27.8%). This change reclassified 33 patients (24.8%) from DUCA-TO non-achievement to achievement, with no reverse reclassification because all other DUCA-TO components were kept unchanged (Table 2).

A sensitivity analysis comparing Original DUCA-TO and Modified DUCA-TO definitions is summarized in Supplementary Table S3.

### 3.3. Patient-Level Concordance and Discordance Between the Evaluated Surgical/Perioperative TOGS Components and DUCA-TO Definitions

When the evaluated surgical/perioperative TOGS components were compared with Original DUCA-TO, patient-level concordance was observed in 105 of 133 patients (78.9%), whereas discordance was observed in 28 patients (21.1%). Cohen’s kappa was 0.563, and McNemar testing demonstrated significant directional asymmetry (*p* < 0.001). Discordance was strongly asymmetric: 26 patients were classified as TOGS-positive but Original DUCA-TO-negative, whereas only 2 patients were classified in the opposite direction. This pattern indicates that Original DUCA-TO produced a markedly more restrictive classification than TOGS.

When the evaluated surgical/perioperative TOGS components were compared with Modified DUCA-TO, concordance increased to 112 of 133 patients (84.2%), and discordance decreased to 21 patients (15.8%). Cohen’s kappa was 0.686, and McNemar testing was no longer statistically significant (*p* = 0.078). Notably, the direction of discordance reversed after modifying the DUCA morbidity threshold: Modified DUCA-TO classified more patients as achievement-positive than TOGS among discordant cases. Specifically, 6 patients were classified as TOGS-positive but Modified DUCA-TO-negative, whereas 15 patients were classified as TOGS-negative but Modified DUCA-TO-positive (Table 3).

### 3.4. Factors Associated with TOGS, Original DUCA-TO, and Modified DUCA-TO Achievement

Univariable factors associated with TOGS, Original DUCA-TO, and Modified DUCA-TO achievement are shown in Supplementary Table S4. In multivariable logistic regression analysis, preoperative hemoglobin was independently associated with both TOGS and Original DUCA-TO achievement. Each 1 g/dL increase in preoperative hemoglobin was associated with higher odds of TOGS achievement (adjusted OR, 1.50; 95% CI, 1.19–1.88; *p* < 0.001). Hemoglobin also remained independently associated with Original DUCA-TO achievement (adjusted OR, 1.67; 95% CI, 1.28–2.19; *p* < 0.001).

Preoperative hemoglobin showed moderate discrimination for TOGS achievement (AUC, 0.697; 95% CI, 0.607–0.782). The Youden-derived cut-off was 12.0 g/dL, with 72.1% sensitivity and 68.1% specificity (Supplementary Figure S1).

The strength of the hemoglobin–TO association varied according to the morbidity threshold. In the DUCA-based sensitivity analysis, the adjusted hemoglobin association attenuated from an adjusted OR of 1.67 (95% CI, 1.28–2.19; *p* < 0.001) in Original DUCA-TO to an adjusted OR of 1.33 (95% CI, 1.07–1.66; *p* = 0.010) in Modified DUCA-TO. This attenuation after removing CD grade II morbidity from the DUCA failure criterion suggests partial definitional reinforcement through transfusion-linked grade II events.

ASA physical status ≥III showed a stronger inverse association with Original DUCA-TO achievement (adjusted OR, 0.16; 95% CI, 0.03–0.77; *p* = 0.022) than with TOGS achievement (adjusted OR, 0.41; 95% CI, 0.16–1.05; *p* = 0.062). Age and neoadjuvant chemotherapy were not independently associated with either TOGS or Original DUCA-TO achievement. In a supplementary ROC analysis, preoperative hemoglobin showed moderate discrimination for Original DUCA-TO achievement (AUC, 0.760; 95% CI, 0.679–0.840), with an optimal Youden cut-off of ≥11.6 g/dL (Supplementary Figure S2). Preoperative hemoglobin showed an AUC of 0.652 (95% CI, 0.554–0.746) for Modified DUCA-TO achievement, with an optimal Youden cut-off of 11.6 g/dL (Supplementary Figure S3). In univariable analyses, greater intraoperative blood loss and greater intraoperative erythrocyte transfusion exposure were associated with lower odds of achievement across all three TO definitions, while postoperative erythrocyte transfusion requirement was associated with lower odds of TOGS and Modified DUCA-TO achievement; the corresponding association for Original DUCA-TO was not estimable because of complete separation (Supplementary Table S4). In separate adjusted models, each 50 mL increase in intraoperative blood loss was associated with lower odds of TOGS achievement (adjusted OR, 0.13; 95% CI, 0.05–0.34; *p* < 0.001) and Original DUCA-TO achievement (adjusted OR, 0.11; 95% CI, 0.03–0.41; *p* = 0.001). Similarly, each 1-unit increase in intraoperative erythrocyte transfusion was associated with lower odds of TOGS achievement (adjusted OR, 0.14; 95% CI, 0.06–0.37; *p* < 0.001) and Original DUCA-TO achievement (adjusted OR, 0.13; 95% CI, 0.03–0.57; *p* = 0.007). Postoperative erythrocyte transfusion requirement was also associated with lower odds of TOGS achievement (adjusted OR, 0.26; 95% CI, 0.11–0.59; *p* = 0.001). The adjusted association between postoperative erythrocyte transfusion and Original DUCA-TO could not be estimated by standard logistic regression because no patient receiving postoperative transfusion achieved Original DUCA-TO, resulting in complete separation. Intraoperative blood loss and intraoperative erythrocyte transfusion were strongly correlated (Spearman ρ = 0.806; *p* < 0.001) and were therefore not entered simultaneously into the same adjusted model (Table 4).

### 3.5. Overall Survival According to Textbook Outcome Achievement

During the follow-up, 95 patients died, and 38 patients were censored alive at last follow-up. The median overall survival for the entire cohort was 23.8 months. The median follow-up calculated using the reverse Kaplan–Meier method was 100.9 months [IQR, 71.2–110.7].

In Kaplan–Meier analysis, TOGS achievement was associated with longer overall survival than TOGS non-achievement (46.8 vs. 14.9 months; log-rank *p* = 0.002). Modified DUCA-TO achievement was also associated with longer overall survival (27.9 vs. 14.9 months; log-rank *p* = 0.009). For Original DUCA-TO, the difference in overall survival did not reach statistical significance (28.4 vs. 20.3 months; log-rank *p* = 0.052).

After adjustment for ASA physical status ≥III, p/ypT ≥3 disease, preoperative hemoglobin, and neoadjuvant chemotherapy, none of the TO definitions remained independently associated with overall survival. TOGS achievement showed a borderline association with a lower hazard of death, but this did not reach statistical significance (adjusted HR, 0.64; 95% CI, 0.40–1.02; *p* = 0.062). Original DUCA-TO achievement (adjusted HR, 0.84; 95% CI, 0.48–1.47; *p* = 0.544) and Modified DUCA-TO achievement (adjusted HR, 0.70; 95% CI, 0.45–1.08; *p* = 0.105) were also not independently associated with overall survival. Across all three adjusted Cox models, p/ypT ≥3 disease remained strongly associated with overall survival (HR range, 3.42–3.64; all *p* ≤ 0.005) (Table 5 and Figure 2, Figure 3 and Figure 4).

## 4. Discussion

In this homogeneous Turkish cohort of patients undergoing open D2 total gastrectomy for gastric adenocarcinoma, the surgical/perioperative TOGS components and Original DUCA-TO produced markedly different achievement rates in the same population. This discrepancy was driven mainly by the more restrictive structure of Original DUCA-TO, particularly its inclusion of CD grade ≥II morbidity as a failure criterion. Changing the DUCA morbidity threshold from CD grade ≥II to ≥III increased DUCA-based achievement, increased patient-level agreement with TOGS, and reclassified 24.8% of patients. These findings indicate that DUCA-TO classification is substantially influenced by morbidity-threshold selection. Preoperative hemoglobin was independently associated with the achievement of both definitions, although its association with Original DUCA-TO requires cautious interpretation because postoperative transfusion contributes to CD grade II morbidity.

Because the present analysis did not include the multimodality-treatment domain of the complete TOGS framework, direct comparison of achievement rates with the published TOGS cohorts should be interpreted cautiously. The 45.9% achievement rate based on the evaluated surgical/perioperative TOGS components in our cohort was numerically close to the 50.2% rate reported in the multicenter GIRCG cohort and was within the broad achievement range reported in the gastrectomy TO literature [16,18,19]. A higher TOGS achievement rate of 74.7% was reported in a high-volume Korean external validation cohort [17]. In that Korean cohort, early lesions, distal gastrectomy, and minimally invasive surgery were associated with TOGS achievement [17]. By contrast, our cohort was restricted to open D2 total gastrectomy and included a high proportion of advanced p/ypT ≥3 disease, representing a more selected and surgically demanding population. These structural differences may explain the lower TOGS rate observed in our study. The most frequent TOGS failure component was inadequate lymph-node retrieval (<25 examined lymph nodes), based on the total gastrectomy-specific threshold of ≥25 examined lymph nodes. This finding is consistent with literature showing that nodal adequacy is a major barrier to TOGS achievement [16,18,19]. The prominence of this component despite D2 lymphadenectomy suggests that TOGS achievement is influenced not only by surgical dissection but also by specimen handling and pathological lymph-node retrieval; therefore, standardized specimen processing may represent a modifiable quality-improvement target.

Original DUCA-TO achievement in our cohort was substantially lower than the 45.7% rate reported for gastric cancer patients in the institutional validation study by van der Kaaij et al. [9]. This lower rate likely reflects cohort selection, including open D2 total gastrectomy only and a high proportion of advanced p/ypT disease, rather than differences in the DUCA-TO definition itself. In our cohort, Original DUCA-TO failure was driven predominantly by CD grade ≥II morbidity, consistent with the same study, in which postoperative complications of grade II or higher were the main limiting factor after gastrectomy [9]. CD grade II includes pharmacological treatment, blood transfusion, and total parenteral nutrition [12]. Accordingly, Original DUCA-TO may be particularly sensitive to treatment-requiring but relatively low-grade postoperative events.

The patient-level comparison between the evaluated surgical/perioperative TOGS components and Original DUCA-TO showed that these composite quality metrics were related but not interchangeable. In our cohort, discordance was strongly asymmetric: 26 patients achieved TOGS but failed Original DUCA-TO, whereas only 2 patients showed the opposite pattern. This indicates that Original DUCA-TO produced a more restrictive classification, largely because of its CD grade ≥II morbidity component. After changing the morbidity threshold from CD grade ≥II to ≥III, patient-level agreement increased and discordance decreased. Importantly, the direction of discordance also reversed: with Modified DUCA-TO, 15 patients were TOGS-negative but Modified DUCA-TO-positive, whereas only 6 patients were TOGS-positive but Modified DUCA-TO-negative. This suggests that TOGS and DUCA-TO differ not merely in strictness but also in the subgroups they classify as TO failures, depending on the morbidity threshold selected.

Raising the Original DUCA-TO morbidity threshold from CD grade ≥II to ≥III increased the achievement rate from 27.8% to 52.6% and reclassified 33 patients (24.8%) from non-achievement to achievement. These findings indicate that DUCA-TO classification is substantially influenced by the selected morbidity threshold. Published gastrectomy TO studies have used heterogeneous definitions, including different morbidity thresholds, limiting direct cross-study comparability [18,19]. The CD grade ≥III-based Modified DUCA-TO should therefore be interpreted as a sensitivity analysis rather than as a replacement for the original DUCA framework. The use of CD grade ≥III or ≥IIIa as postoperative morbidity components in gastrectomy-specific TO studies provides methodological precedent for this sensitivity analysis [13,14,15]. Postoperative blood transfusion is classified as a CD grade II complication [12]. Accordingly, associations involving postoperative transfusion should be interpreted with consideration of possible definitional overlap. In multivariable analyses, ASA physical status ≥III showed a stronger inverse association with Original DUCA-TO than with TOGS, suggesting greater sensitivity of the former to perioperative frailty and treatment-requiring postoperative events. Preoperative hemoglobin was independently associated with achievement of both definitions. In additional exploratory analyses, greater intraoperative blood loss and higher intraoperative erythrocyte transfusion requirements were each associated with lower odds of TO achievement in separately adjusted models. The hemoglobin association remained statistically significant for both TOGS and Original DUCA-TO in models additionally adjusted for intraoperative blood loss; however, these exploratory analyses do not establish the independent mechanistic contributions of preoperative hemoglobin and operative blood loss. Because intraoperative blood loss and erythrocyte transfusion were strongly correlated, their individual contributions could not be clearly separated in this cohort. These observations are consistent with gastrectomy literature linking preoperative hemoglobin, anemia, and perioperative transfusion with TO achievement and adverse postoperative outcomes [18,20,21]. The association between hemoglobin and Original DUCA-TO nevertheless requires cautious interpretation because postoperative erythrocyte transfusion contributes to CD grade II morbidity. None of the 50 patients with CD grade II morbidity and postoperative erythrocyte transfusion achieved Original DUCA-TO, and the hemoglobin association attenuated after removal of CD grade II morbidity from the DUCA failure definition. Postoperative erythrocyte transfusion was also associated with lower odds of TOGS achievement, whereas its association with Original DUCA-TO was not estimable by standard logistic regression because of complete separation. Accordingly, preoperative hemoglobin may be best regarded as an easily reportable perioperative risk marker rather than a standalone causal biomarker or decision-making tool for TO achievement.

Survival analyses revealed a discrepancy between unadjusted and adjusted prognostic associations for all TO definitions. TOGS and Modified DUCA-TO achievement were associated with longer overall survival in unadjusted analyses, whereas the corresponding difference for Original DUCA-TO did not reach statistical significance. However, no TO definition remained independently associated with overall survival after adjustment for ASA physical status, p/ypT ≥3 disease, preoperative hemoglobin, and neoadjuvant chemotherapy. The relatively short median overall survival of 23.8 months should be interpreted in the context of the advanced disease burden of this cohort; among patients with retrievable pretreatment staging data, 90.6% had cT3–4 disease and 83.5% had cN-positive disease. Longer survival estimates have been reported in previous gastrectomy cohorts [9,17]. Previous studies have also reported associations between TO achievement and longer-term survival after gastric cancer surgery [9,10,11,17]. In our cohort, p/ypT ≥3 disease remained strongly associated with overall survival across all adjusted Cox models, whereas the associations between TO achievement and overall survival were attenuated after adjustment. Given the retrospective observational design, these findings should not be interpreted as evidence of a causal effect of TO achievement on survival and should be considered exploratory and hypothesis-generating.

### Limitations

Several limitations should be acknowledged. First, the retrospective single-center design and the modest cohort size (n = 133) may have limited statistical power, particularly for multivariable and subgroup analyses, and reduced the precision of some effect estimates; these factors may also limit the generalizability of the findings. Moreover, the exclusion of patients with insufficient historical records may have introduced selection bias. Second, restricting the cohort to open D2 total gastrectomy improved procedural homogeneity but limits generalizability to minimally invasive approaches and to subtotal or proximal gastrectomy populations. Third, the multimodality-treatment domain of the full TOGS framework was outside the scope of this study; therefore, our analysis reflects surgical/perioperative TOGS components rather than the full TOGS framework or textbook oncological outcomes. The CD grade ≥III threshold used in Modified DUCA-TO was examined as part of a sensitivity analysis and should not be interpreted as a validated replacement for the original DUCA-TO definition. Fourth, pretreatment clinical T and N categories were retrievable for only 85 patients (63.9%) and were therefore summarized descriptively rather than included in the adjusted models, limiting comprehensive adjustment for pretreatment tumor burden. Fifth, curative intent and radicality at the end of surgery were determined retrospectively from operative records, which may have introduced documentation-related misclassification. Furthermore, the retrospective observational design precludes causal interpretation of the observed association between TO achievement and overall survival. Finally, the limited number of Original DUCA-TO achievers and small subgroup counts, particularly for ASA ≥III, further reduced the precision of some regression estimates; therefore, the regression and survival analyses should be interpreted as exploratory.

## 5. Conclusions

In this homogeneous cohort of patients undergoing open D2 total gastrectomy for gastric adenocarcinoma, the evaluated surgical/perioperative TOGS components and DUCA-based TO definitions were related but not interchangeable. The DUCA morbidity threshold substantially influenced classification behavior, and removing CD grade II morbidity changed achievement rates, the direction of discordance, and the interpretation of the hemoglobin association. Future TO studies should explicitly report the definitions used, morbidity thresholds, and component-level failure patterns to improve cross-cohort comparability. Multicenter prospective validation is needed to determine whether these definition-dependent differences persist across different surgical volumes, case mixes, and perioperative care systems.

## Figures and Tables

**Figure 1:**
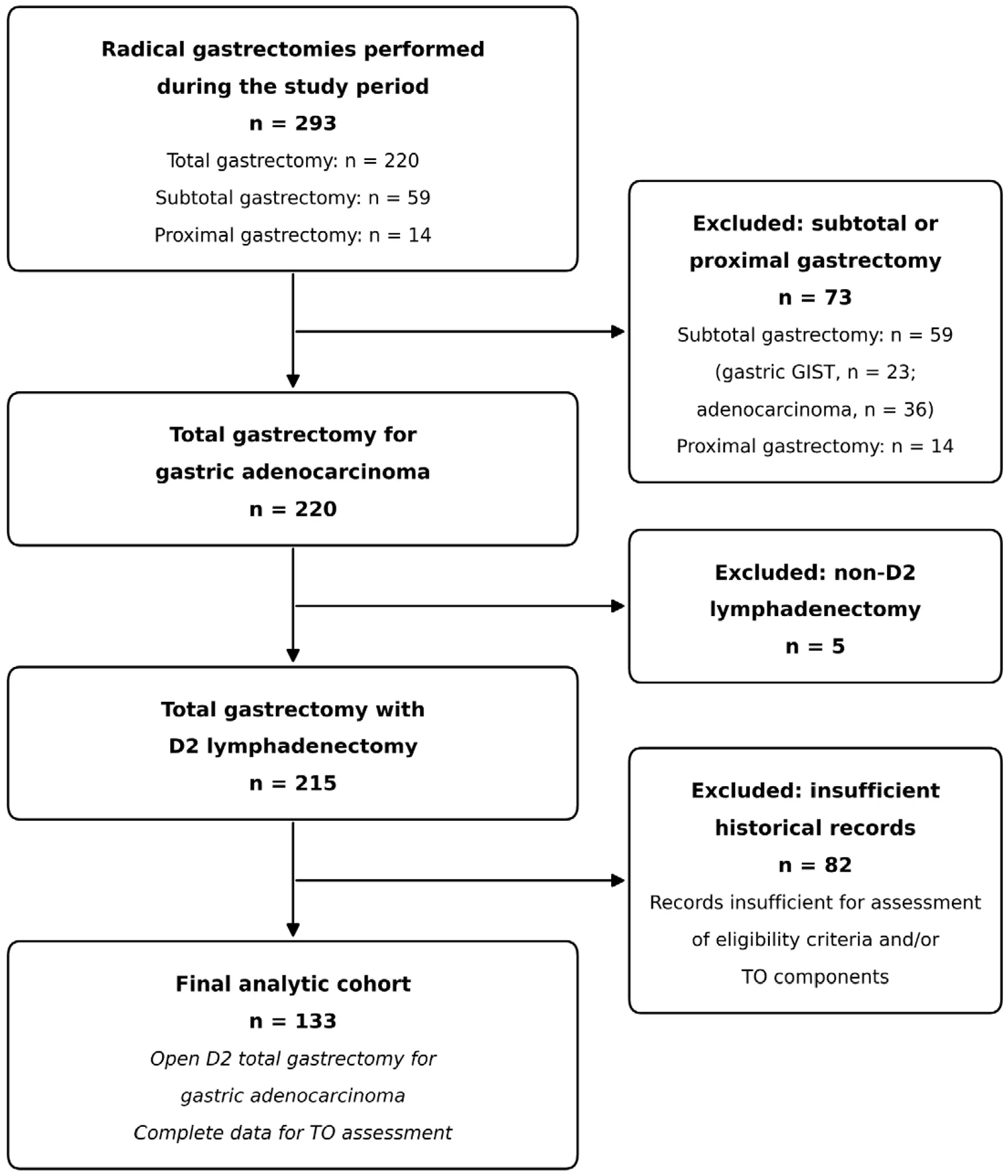
Flow diagram of patient selection.

**Figure 2:**
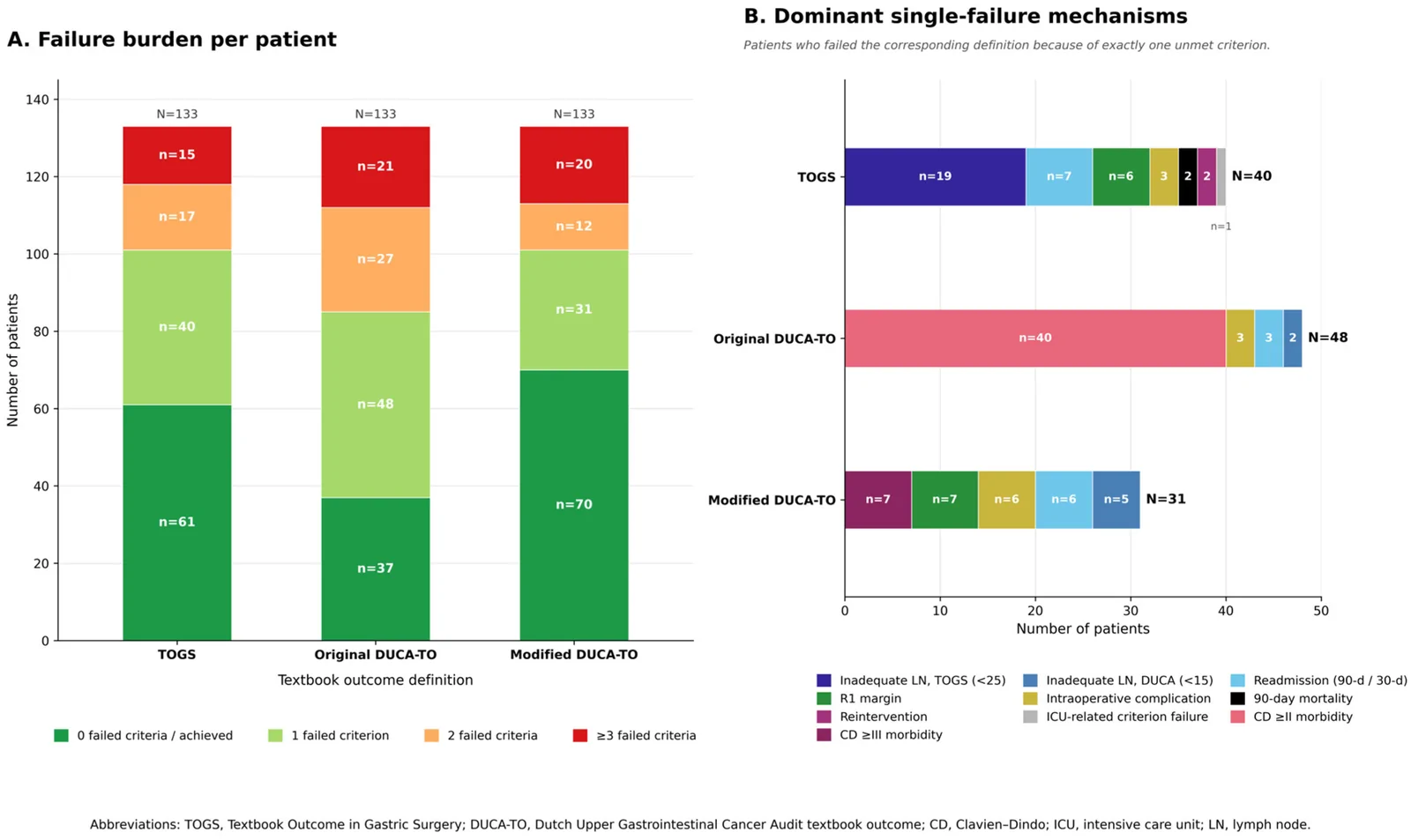
Patient-level failure burden and dominant single-failure mechanisms across TOGS, Original DUCA-TO, and Modified DUCA-TO definitions. Panel (**A**) shows the distribution of the number of failed criteria per patient for each TO definition. Patients with zero failed criteria were classified as achieving the corresponding outcome. Panel (**B**) shows the dominant single-failure mechanisms among patients who failed each definition because of only one unmet criterion. The ICU-related criterion was defined according to the corresponding TO framework: for TOGS, as unplanned ICU/medium-care unit admission for a life-threatening complication within 30 days after surgery, and for Original and Modified DUCA-TO, as readmission to the ICU within 30 days after surgery. For TOGS, hospital readmission was defined as complication-related readmission within 90 days after surgery and mortality as death during the initial hospital stay and/or within 90 days after surgery. For DUCA-TO definitions, hospital readmission was assessed within 30 days after discharge and mortality as postoperative mortality within 30 days after surgery. Original DUCA-TO was calculated using CD grade ≥II morbidity as failure, whereas Modified DUCA-TO used CD grade ≥III morbidity as failure, with all other DUCA-TO components unchanged. R1 margin indicates a tumor-positive microscopic resection margin; no patient failed the DUCA potentially curative/radical resection component. Component failures are not mutually exclusive except in the single-failure subgroup. TOGS refers only to the evaluated surgical/perioperative components of the GIRCG framework; the multimodality-treatment domain was not assessed. TOGS, Textbook Outcome in Gastric Surgery; DUCA-TO, Dutch Upper Gastrointestinal Cancer Audit textbook outcome; CD, Clavien–Dindo; ICU, intensive care unit; LN, lymph node.

**Figure 3:**
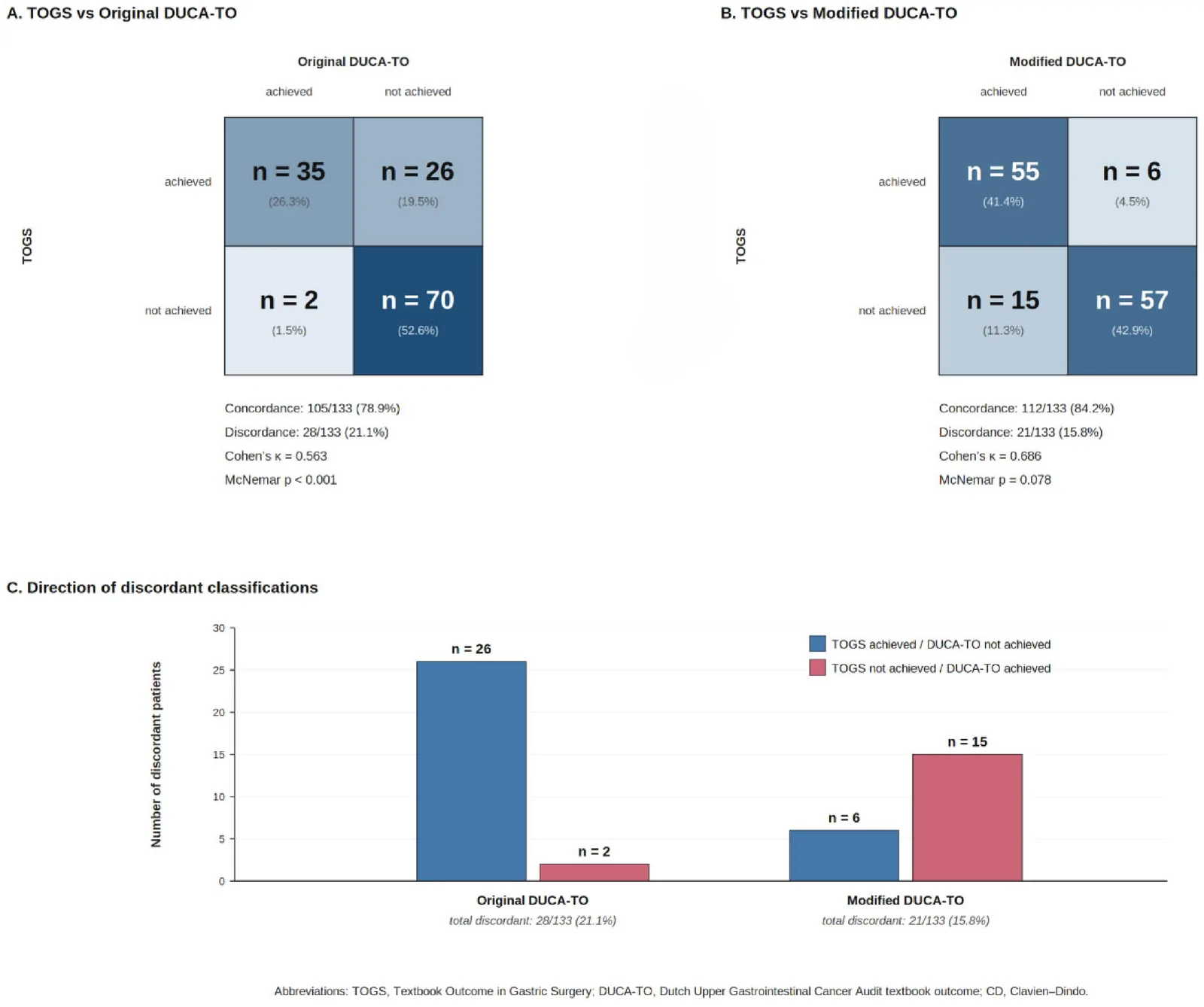
Patient-level concordance matrices and discordance patterns between TOGS and DUCA-TO definitions. Panel (**A**) shows the concordance matrix between TOGS and Original DUCA-TO using CD grade ≥II morbidity as failure. Panel (**B**) shows the concordance matrix between TOGS and Modified DUCA-TO using CD grade ≥III morbidity as failure, while all other DUCA-TO components were kept unchanged. Panel (**C**) illustrates the direction and magnitude of discordant classifications between definitions. Concordance was defined as classification of the same patient as achievement-positive or achievement-negative by both definitions; discordance was defined as disagreement between definitions for the same patient. TOGS refers only to the evaluated surgical/perioperative components of the GIRCG framework; the multimodality-treatment domain was not assessed. TOGS, Textbook Outcome in Gastric Surgery; DUCA-TO, Dutch Upper Gastrointestinal Cancer Audit textbook outcome; CD, Clavien–Dindo.

**Figure 4:**
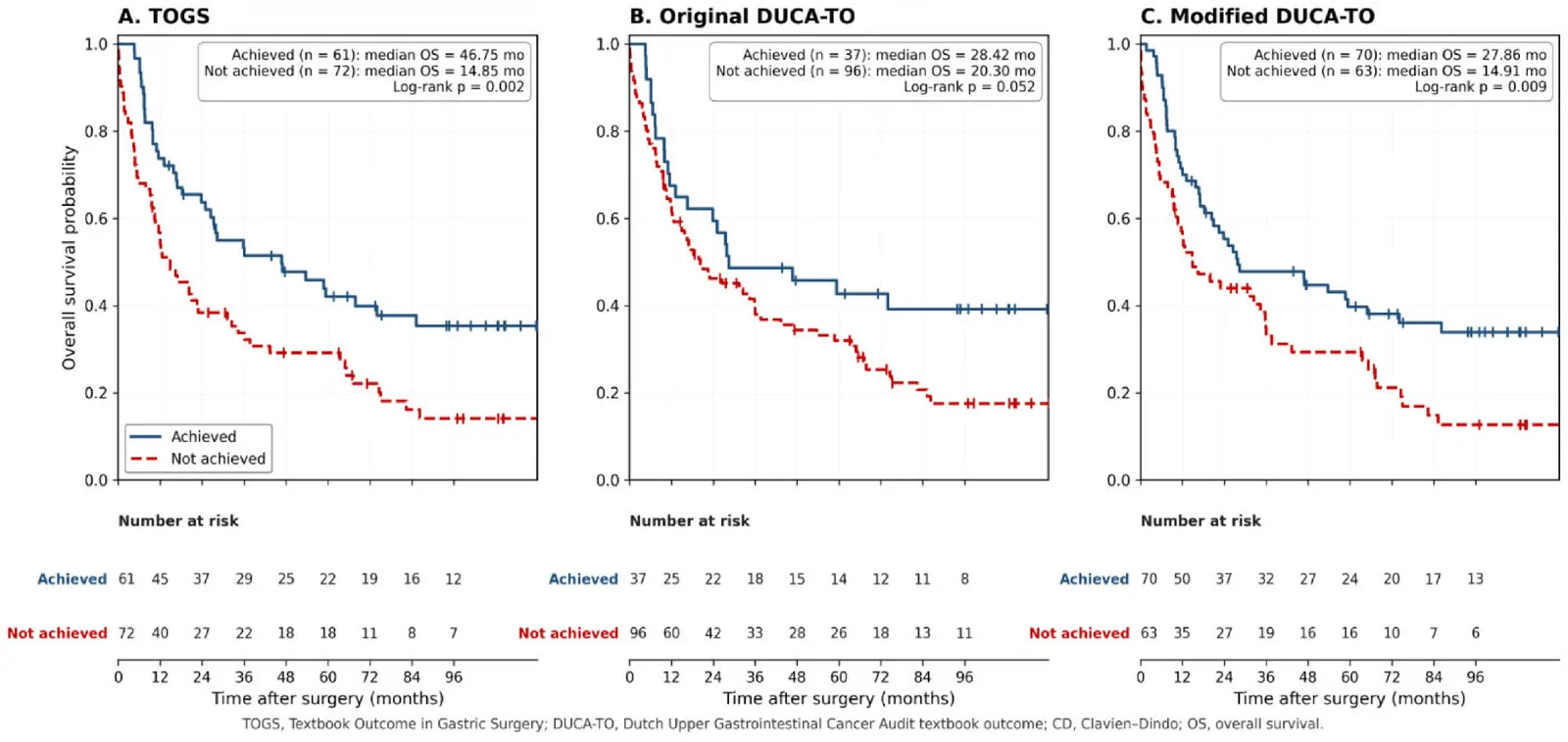
Kaplan–Meier overall survival curves according to textbook outcome achievement. Panel (**A**) shows overall survival according to TOGS achievement. Panel (**B**) shows overall survival according to Original DUCA-TO achievement using CD grade ≥II morbidity as failure. Panel (**C**) shows overall survival according to Modified DUCA-TO achievement using CD grade ≥III morbidity as failure, while all other DUCA-TO components were kept unchanged. Survival curves were compared using the log-rank test. Number at risk is shown below each panel. TOGS refers only to the evaluated surgical/perioperative components of the GIRCG framework; the multimodality-treatment domain was not assessed. TOGS, Textbook Outcome in Gastric Surgery; DUCA-TO, Dutch Upper Gastrointestinal Cancer Audit textbook outcome; CD, Clavien–Dindo; OS, overall survival.

**Table 1:** Baseline characteristics of the overall cohort.

Variable	Overall Cohort, *N* = 133
Age, years	64 [56–71]
Male sex, *n* (%)	85 (63.9)
ASA physical status ≥III, *n* (%)	33 (24.8)
Neoadjuvant chemotherapy, *n* (%)	60 (45.1)
Pretreatment cT3–4 disease, *n*/*N* (%)	77/85 (90.6)
Pretreatment cN-positive disease, *n*/*N* (%)	71/85 (83.5)
Diffuse Lauren type, *n* (%)	61 (45.9)
High grade, G3–G4, *n* (%)	73 (54.9)
p/ypT ≥3 disease, *n* (%)	115 (86.5)
p/ypN-positive disease, *n* (%)	93 (69.9)
Linitis plastica, *n* (%)	12 (9.0)
Hemoglobin, g/dL	12.0 [10.3–12.5]
Preoperative anemia, *n* (%)	88 (66.2)
Albumin, g/L	34.0 [28.0–37.7]

Values are presented as median [interquartile range] or *n* (%). All patients underwent open D2 total gastrectomy for gastric adenocarcinoma. Pathological T and N categories were reported as pT/pN after upfront surgery and as ypT/ypN after neoadjuvant chemotherapy. Accordingly, p/ypT and p/ypN variables refer to the applicable pathological stage according to treatment sequence. Pretreatment clinical T and N percentages were calculated among the 85 patients with retrievable preoperative staging data. High grade was defined as G3–G4. Preoperative anemia was defined as hemoglobin <13.0 g/dL in men and <12.0 g/dL in women. ASA, American Society of Anesthesiologists;

**Table 2:** Achievement rates and component-level failures of the evaluated surgical/perioperative TOGS components, Original DUCA-TO, and Modified DUCA-TO.

Outcome/Component	TOGS	Original DUCA-TO CD ≥II	Modified DUCA-TO CD ≥III
Achievement status			
Achieved, *n* (%)	61 (45.9)	37 (27.8)	70 (52.6)
Not achieved, *n* (%)	72 (54.1)	96 (72.2)	63 (47.4)
Component-level failure			
Morbidity threshold failure, *n* (%)	—	87 (65.4)	37 (27.8)
Potentially curative/radical resection not achieved, *n* (%)	—	0 (0.0)	0 (0.0)
Tumor-negative resection margin not achieved/R1, *n* (%)	20 (15.0)	20 (15.0)	20 (15.0)
Intraoperative complication, *n* (%)	13 (9.8)	13 (9.8)	13 (9.8)
Inadequate lymph-node retrieval, *n* (%)	41 (30.8)	13 (9.8)	13 (9.8)
Reintervention, *n* (%)	13 (9.8)	13 (9.8)	13 (9.8)
ICU-related criterion failure, *n* (%)	4 (3.0)	4 (3.0)	4 (3.0)
Readmission, *n* (%)	20 (15.0)	14 (10.5)	14 (10.5)
Mortality, *n* (%)	17 (12.8)	11 (8.3)	11 (8.3)
Prolonged length of stay >21 days, *n* (%)	—	8 (6.0)	8 (6.0)

Values are presented as *n* (%). TOGS was evaluated using the applicable surgical and perioperative components of the GIRCG TOGS framework; CD grade and the multimodality-treatment domain of the full TOGS framework were not included. For TOGS, inadequate lymph-node retrieval was defined as <25 examined lymph nodes for total gastrectomy, ICU-related criterion failure as unplanned ICU/medium-care unit admission for a life-threatening complication within 30 days after surgery, hospital readmission as complication-related readmission within 90 days after surgery, and mortality as death during the initial hospital stay and/or within 90 days after surgery. Original and Modified DUCA-TO used CD grade ≥II and ≥III morbidity as failure criteria, respectively; all other DUCA-TO components were unchanged. For DUCA-TO, inadequate lymph-node retrieval was defined as <15 examined lymph nodes, ICU-related criterion failure as readmission to the ICU within 30 days after surgery, prolonged length of stay as >21 days, hospital readmission as readmission within 30 days after discharge, and mortality as postoperative mortality within 30 days after surgery. Potentially curative/radical resection was determined from operative records; no patient failed this component. Component failures were not mutually exclusive. TOGS, Textbook Outcome in Gastric Surgery; DUCA-TO, Dutch Upper Gastrointestinal Cancer Audit textbook outcome; GIRCG, Italian Gastric Cancer Research Group; CD, Clavien–Dindo; ICU, intensive care unit.

**Table 3:** Patient-level concordance and discordance analysis between the evaluated surgical/perioperative TOGS components and DUCA-TO definitions.

Analysis	Result
A. TOGS vs. Original DUCA-TO CD ≥II	
Concordant classification, *n* (%)	105 (78.9)
Discordant classification, *n* (%)	28 (21.1)
Cohen’s kappa	0.563
McNemar test, *p* value	<0.001
TOGS+/Original DUCA-TO+, *n*	35
TOGS+/Original DUCA-TO−, *n*	26
TOGS−/Original DUCA-TO+, *n*	2
TOGS−/Original DUCA-TO−, *n*	70
B. TOGS vs. Modified DUCA-TO CD ≥III	
Concordant classification, *n* (%)	112 (84.2)
Discordant classification, *n* (%)	21 (15.8)
Cohen’s kappa	0.686
McNemar test, *p* value	0.078
TOGS+/Modified DUCA-TO+, *n*	55
TOGS+/Modified DUCA-TO−, *n*	6
TOGS−/Modified DUCA-TO+, *n*	15
TOGS−/Modified DUCA-TO−, *n*	57

Values are presented as *n* (%), unless otherwise indicated. TOGS refers to the evaluated surgical and perioperative components of the GIRCG TOGS framework; the multimodality-treatment domain was not assessed. TOGS+ and DUCA-TO+ indicate achievement of the corresponding TO definition; TOGS− and DUCA-TO− indicate non-achievement. Original DUCA-TO was calculated using CD grade ≥II morbidity as failure. Modified DUCA-TO was calculated using CD grade ≥III morbidity as failure, while all other DUCA-TO components were kept unchanged. Concordance was defined as classification of the same patient as either achievement-positive by both definitions or achievement-negative by both definitions. Discordance was defined as disagreement between definitions for the same patient. Cohen’s kappa was used to quantify chance-corrected agreement between definitions. McNemar testing was used to evaluate directional asymmetry in paired classifications. TOGS, Textbook Outcome in Gastric Surgery; DUCA-TO, Dutch Upper Gastrointestinal Cancer Audit textbook outcome; CD, Clavien–Dindo.

**Table 4:** Multivariable logistic regression analyses for TOGS and Original DUCA-TO achievement.

Variable	TOGS Achievement Adjusted OR (95% CI)	*p* Value	Original DUCA-TO Achievement Adjusted OR (95% CI)	*p* Value
Age, per year	1.00 (0.98–1.03)	0.791	0.99 (0.96–1.02)	0.591
ASA physical status ≥III	0.41 (0.16–1.05)	0.062	0.16 (0.03–0.77)	0.022
Hemoglobin, per 1 g/dL	1.50 (1.19–1.88)	<0.001	1.67 (1.28–2.19)	<0.001
Neoadjuvant chemotherapy	1.25 (0.59–2.64)	0.567	1.33 (0.56–3.17)	0.518
Intraoperative blood loss, per 50 mL increase †	0.13 (0.05–0.34)	<0.001	0.11 (0.03–0.41)	0.001
Intraoperative erythrocyte transfusion, per 1-unit increase †	0.14 (0.06–0.37)	<0.001	0.13 (0.03–0.57)	0.007
Postoperative erythrocyte transfusion requirement †	0.26 (0.11–0.59)	0.001	Not estimable ‡	—

Values are adjusted odds ratios (ORs) with 95% confidence intervals (CIs). Primary models included age, ASA physical status ≥III, preoperative hemoglobin, and neoadjuvant chemotherapy. † Each additional exploratory variable was evaluated in a separate model adjusted for the same four covariates. Intraoperative blood loss and intraoperative erythrocyte transfusion were not entered simultaneously because of strong correlation. ‡ Postoperative erythrocyte transfusion was not estimable for Original DUCA-TO because of complete separation. OR >1 indicates higher odds of TO achievement. TOGS refers to the evaluated surgical/perioperative components of the GIRCG TOGS framework; the multimodality-treatment domain was not assessed. ASA, American Society of Anesthesiologists; DUCA-TO, Dutch Upper Gastrointestinal Cancer Audit textbook outcome; TOGS, Textbook Outcome in Gastric Surgery.

**Table 5:** Survival outcomes and Cox regression summary according to textbook outcome achievement.

Survival Outcome	TOGS	Original DUCA-TO	Modified DUCA-TO
Achieved/not achieved, *n*	61/72	37/96	70/63
Median OS, months, achieved vs. not achieved	46.8 vs. 14.9	28.4 vs. 20.3	27.9 vs. 14.9
Log-rank *p* value	0.002	0.052	0.009
Multivariable HR for achievement (95% CI)	0.64 (0.40–1.02)	0.84 (0.48–1.47)	0.70 (0.45–1.08)
Multivariable *p* value	0.062	0.544	0.105

Values are presented as median overall survival in months or hazard ratios with 95% confidence intervals. Multivariable Cox proportional hazards models were adjusted for ASA physical status ≥III, p/ypT ≥3 disease, preoperative hemoglobin, and neoadjuvant chemotherapy. The hazard ratio refers to achievement of the corresponding TO definition compared with non-achievement. HR <1 indicates a lower hazard of death. Detailed Cox regression models including all covariates are presented in Supplementary Table S5. TOGS refers to the evaluated surgical/perioperative components of the GIRCG TOGS framework; the multimodality-treatment domain was not assessed. CI, confidence interval; DUCA-TO, Dutch Upper Gastrointestinal Cancer Audit textbook outcome; HR, hazard ratio; OS, overall survival; TOGS, Textbook Outcome in Gastric Surgery.

## Data Availability

The data presented in this study are available on request from the corresponding author due to privacy and ethical restrictions related to patient records.

## Supplementary Materials

The following supporting information can be downloaded at: https://www.mdpi.com/article/10.3390/medicina62101854/s1, Table S1: Selected non-component clinical, pathological, laboratory, and operative characteristics stratified by TOGS achievement; Table S2: Selected non-component clinical, pathological, laboratory, and operative characteristics stratified by Original DUCA-TO achievement; Table S3: Sensitivity analysis comparing Original DUCA-TO and Modified DUCA-TO definitions; Table S4: Univariable factors associated with TOGS, Original DUCA-TO, and Modified DUCA-TO achievement; Table S5: Detailed multivariable Cox proportional hazards regression models for overall survival; Figure S1: Receiver operating characteristic curve of preoperative hemoglobin for predicting TOGS achievement; Figure S2: Receiver operating characteristic curve of preoperative hemoglobin for predicting Original DUCA-TO achievement; Figure S3: Receiver operating characteristic curve of preoperative hemoglobin for predicting Modified DUCA-TO achievement.
