# Supplementary material for: Patient-Level Comparison of Surgical TOGS Components and DUCA-Based Textbook Outcome After Open D2 Total Gastrectomy
Source: Medicina (Kaunas). 2026 Sep 24;62(10):1854. doi: 10.3390/medicina62101854 (PMC13707275; doi:10.3390/medicina62101854)
Supplement: Supplementary file 1 [file medicina-62-01854-s001.zip › medicina-4523182-supplementary.pdf]

Supplementary Materials

Supplementary Table S1. Selected non-component clinical, pathological, laboratory, and operative characteristics stratified by TOGS achievement

Variable	TOGS achieved, n=61	TOGS not achieved, n=72	p value
**Clinical characteristics**			
**BMI, kg/m²**	23.9 [21.3–26.3]	24.0 [22.5–26.0]	0.897
**CCI total score**	2 [2–3]	3 [2–3]	0.481
**ASA physical status ≥III, n (%)**	9 (14.8)	24 (33.3)	0.013
**Neoadjuvant chemotherapy, n (%)**	28 (45.9)	32 (44.4)	0.866
**Pathological characteristics**			
**p/ypT ≥3 disease, n (%)**	47 (77.0)	68 (94.4)	0.003
**p/ypN-positive disease, n (%)**	38 (62.3)	55 (76.4)	0.077
**Tumor size, cm**	4.0 [2.5–6.0]	5.0 [4.2–7.0]	0.019
**Laboratory parameters**			
**Hemoglobin, g/dL**	12.0 [11.1–13.3]	11.0 [10.0–12.0]	<0.001
**Albumin, g/L**	34.5 [28.0–39.5]	33.2 [27.6–36.1]	0.186
**Operative variable**			
**Operation time, min**	245 [220–300]	235 [195–270]	0.067

Values are presented as median [interquartile range] or n (%). Continuous variables were compared using the Mann–Whitney U test. Categorical variables were compared using the chi-square test or Fisher's exact test, as appropriate. This table includes selected non-component variables and does not include variables that directly constitute TOGS achievement or failure. Pathological T and N categories were reported as pT/pN after upfront surgery and as ypT/ypN after neoadjuvant chemotherapy. Accordingly, p/ypT and p/ypN variables refer to the applicable pathological stage according to treatment sequence. Pretreatment clinical T and N categories were not included because they were retrievable for only 85 patients (63.9%) and were therefore summarized descriptively in the main manuscript. Tumor size was summarized among patients with measurable discrete tumors on final pathology; cases with linitis plastica were recorded as a separate pathological variable. P values are exploratory and were not adjusted for multiple comparisons. TOGS refers to the evaluated surgical/perioperative components of the GIRCG TOGS framework; the multimodality-treatment domain was not assessed. TOGS, Textbook Outcome in Gastric Surgery; BMI, body mass index; CCI, Charlson Comorbidity Index; ASA, American Society of Anesthesiologists.

Supplementary Table S2. Selected non-component clinical, pathological, laboratory, and operative characteristics stratified by Original DUCA-TO achievement

Variable	Original DUCA-TO achieved, n=37	Original DUCA-TO not achieved, n=96	p value
**Clinical characteristics**			
**BMI, kg/m²**	23.8 [20.6–26.0]	24.0 [22.5–26.1]	0.300
**CCI total score**	2 [2–3]	3 [2–3]	0.097
**ASA physical status ≥III, n (%)**	2 (5.4)	31 (32.3)	0.001
**Neoadjuvant chemotherapy, n (%)**	17 (45.9)	43 (44.8)	0.905
**Pathological characteristics**			
**p/ypT ≥3 disease, n (%)**	27 (73.0)	88 (91.7)	0.005
**p/ypN-positive disease, n (%)**	23 (62.2)	70 (72.9)	0.226
**Tumor size, cm**	3.0 [2.4–6.0]	5.0 [4.0–7.0]	0.041
**Laboratory parameters**			
**Hemoglobin, g/dL**	12.2 [12.0–14.0]	11.1 [10.0–12.2]	<0.001
**Albumin, g/L**	34.0 [27.0–39.2]	34.0 [29.0–37.0]	0.912
**Operative variable**			
**Operation time, min**	255 [225–300]	240 [200–280]	0.263

Values are presented as median [interquartile range] or n (%). Continuous variables were compared using the Mann–Whitney U test. Categorical variables were compared using the chi-square test or Fisher's exact test, as appropriate. Original DUCA-TO was calculated using Clavien–Dindo grade ≥II morbidity as failure. This table includes selected non-component variables and does not include variables that directly constitute DUCA-TO achievement or failure. Pathological T and N categories were reported as pT/pN after upfront surgery and as ypT/ypN after neoadjuvant chemotherapy. Accordingly, p/ypT and p/ypN variables refer to the applicable pathological stage according to treatment sequence. Pretreatment clinical T and N categories were not included because they were retrievable for only 85 patients (63.9%) and were therefore summarized descriptively in the main manuscript. Tumor size was summarized among patients with measurable discrete tumors on final pathology; cases with linitis plastica were recorded as a separate pathological variable. P values are exploratory and were not adjusted for multiple comparisons. DUCA-TO, Dutch Upper Gastrointestinal

Cancer Audit textbook outcome; BMI, body mass index; CCI, Charlson Comorbidity Index; ASA, American Society of Anesthesiologists.

Supplementary Table S3. Sensitivity analysis comparing Original DUCA-TO and Modified DUCA-TO definitions

Variable	Original DUCA-TO CD ≥II	Modified DUCA-TO CD ≥III
**Definition-level outcome**		
**Achieved, n (%)**	37 (27.8)	70 (52.6)
**Not achieved, n (%)**	96 (72.2)	63 (47.4)
**Absolute increase in achievement after CD threshold modification**	Reference	+33 patients (+24.8 percentage points)
**Relative increase in achieved cases**	Reference	+89.2%
**Patients reclassified from DUCA-TO negative to DUCA-TO positive, n (%)**	—	33 (24.8)
**Patients reclassified from DUCA-TO positive to DUCA-TO negative, n (%)**	—	0 (0.0)
**Morbidity-threshold effect**		
**Morbidity threshold failure, n (%)**	87 (65.4)	37 (27.8)
**Reduction in morbidity-threshold failure after CD modification**	Reference	−50 patients (−37.6 percentage points)
**Relationship with TOGS**		
**Concordance with TOGS, n (%)**	105 (78.9)	112 (84.2)
**Discordance with TOGS, n (%)**	28 (21.1)	21 (15.8)
**Cohen's kappa with TOGS**	0.563	0.686
**McNemar p value**	<0.001	0.078
**Hemoglobin association**		
**Hemoglobin, univariable OR per 1 g/dL increase**	1.75 (95% CI, 1.35–2.28)	1.38 (95% CI, 1.11–1.70)

**Univariable p value**	<0.001	0.003
**Hemoglobin, adjusted OR per 1 g/dL increase**	1.67 (95% CI, 1.28–2.19)	1.33 (95% CI, 1.07–1.66)
**Adjusted p value**	<0.001	0.010

Values are presented as n (%), odds ratios with 95% confidence intervals, or p values, unless otherwise indicated. Original DUCA-TO used Clavien–Dindo grade ≥II morbidity as failure, whereas Modified DUCA-TO used grade ≥III morbidity as failure; all other DUCA-TO components were unchanged. Reclassification indicates patients whose DUCA-TO status changed after modifying the morbidity threshold. Concordance, discordance, Cohen's kappa, and McNemar tests were calculated against classification based on the evaluated surgical/perioperative TOGS components. The multimodality-treatment domain of the complete TOGS framework was not assessed. Hemoglobin odds ratios are expressed per 1-g/dL increase; adjusted models included age, ASA physical status ≥III, preoperative hemoglobin, and neoadjuvant chemotherapy. DUCA-TO, Dutch Upper Gastrointestinal Cancer Audit textbook outcome; TOGS, Textbook Outcome in Gastric Surgery; CD, Clavien–Dindo; OR, odds ratio; CI, confidence interval; ASA, American Society of Anesthesiologists.

Table S4. Univariable factors associated with TOGS, Original DUCA-TO, and Modified DUCA-TO achievement

Predictor	TOGS achievement OR (95% CI)	p value	Original DUCA-TO achievement OR (95% CI)	p value	Modified DUCA-TO achievement OR (95% CI)	p value
**Age, per year**	0.99 (0.96–1.01)	0.284	0.97 (0.95–1.00)	0.045	0.98 (0.96–1.01)	0.224
**Male sex**	1.49 (0.73–3.06)	0.276	1.77 (0.77–4.07)	0.180	1.18 (0.58–2.40)	0.648
**BMI, per kg/m²**	1.00 (0.91–1.10)	0.977	0.95 (0.85–1.05)	0.309	1.03 (0.94–1.13)	0.543
**CCI total score, per point**	0.88 (0.68–1.13)	0.309	0.79 (0.57–1.10)	0.162	0.72 (0.54–0.95)	0.019
**ASA physical status ≥III**	0.35 (0.15–0.82)	0.016	0.12 (0.03–0.53)	0.005	0.29 (0.12–0.67)	0.004
**Neoadjuvant chemotherapy**	1.06 (0.53–2.10)	0.866	1.05 (0.49–2.24)	0.905	1.19 (0.60–2.36)	0.620
**Diffuse Lauren type**	0.79 (0.40–1.56)	0.490	1.00 (0.47–2.15)	0.991	0.99 (0.50–1.95)	0.971
**High grade, G3–G4**	0.83 (0.42–1.66)	0.605	0.95 (0.45–2.04)	0.905	1.07 (0.54–2.13)	0.840
**p/ypT ≥3 disease**	0.20 (0.06–0.64)	0.007	0.25 (0.09–0.68)	0.007	0.38 (0.13–1.13)	0.081

**p/ypN-positive disease**	0.51 (0.24–1.08)	0.079	0.61 (0.27–1.36)	0.228	0.65 (0.31–1.38)	0.266
**p/ypN3 category**	0.65 (0.32–1.31)	0.226	0.70 (0.32–1.56)	0.385	0.54 (0.26–1.09)	0.085
**Linitis plastica**	0.56 (0.16–1.96)	0.366	0.21 (0.03–1.72)	0.148	1.29 (0.39–4.29)	0.679
**Hemoglobin, per 1 g/dL**	1.53 (1.22–1.92)	<0.001	1.75 (1.35–2.28)	<0.001	1.38 (1.11–1.70)	0.003
**Albumin, per 1 g/L**	1.04 (0.98–1.10)	0.212	0.99 (0.93–1.06)	0.777	1.05 (0.99–1.11)	0.102
**Intraoperative blood loss, per 50-mL increase**	0.11 (0.04–0.27)	<0.001	0.08 (0.02–0.27)	<0.001	0.24 (0.12–0.45)	<0.001
**Intraoperative erythrocyte transfusion, per 1-unit increase**	0.10 (0.04–0.25)	<0.001	0.07 (0.02–0.30)	<0.001	0.15 (0.07–0.32)	<0.001
**Postoperative erythrocyte transfusion requirement**	0.19 (0.09–0.39)	<0.001	Not estimable†	—	0.30 (0.15–0.63)	0.002

Values are odds ratios with 95% confidence intervals from univariable logistic regression models for achievement of the corresponding textbook outcome definition. OR >1 indicates higher odds of achievement. Intraoperative blood loss was modeled per 50-mL increase, intraoperative erythrocyte transfusion per 1-unit increase, and postoperative erythrocyte transfusion requirement as a binary variable. Original DUCA-TO used Clavien–Dindo grade ≥II postoperative morbidity as failure; Modified DUCA-TO used grade ≥III, with all other DUCA-TO components unchanged. The association between postoperative erythrocyte transfusion and Original DUCA-TO was not estimable by standard logistic regression because no patient receiving postoperative transfusion achieved Original DUCA-TO, resulting in complete separation. p/ypT and p/ypN refer to pathological stage after upfront surgery or neoadjuvant chemotherapy, as applicable. p values are exploratory and were not adjusted for multiple comparisons. TOGS refers to the evaluated surgical/perioperative components of the GIRCG TOGS framework; the multimodality-treatment domain was not assessed. TOGS, Textbook Outcome in Gastric Surgery; DUCA-TO, Dutch Upper Gastrointestinal Cancer Audit textbook outcome; OR, odds ratio; CI, confidence interval; BMI, body mass index; CCI, Charlson Comorbidity Index; ASA, American Society of Anesthesiologists.

Supplementary Table S5. Detailed multivariable Cox proportional hazards regression models for overall survival

Variable	HR	95% CI	p value
**A. TOGS Cox model**			
**TOGS achieved**	0.64	0.40–1.02	0.062
**ASA physical status ≥III**	1.76	1.11–2.81	0.017

**p/ypT ≥3 disease**	3.42	1.44–8.15	0.005
**Hemoglobin, per 1 g/dL**	1.07	0.94–1.21	0.329
**Neoadjuvant chemotherapy**	0.99	0.65–1.50	0.964
**B. Original DUCA-TO Cox model**			
**Original DUCA-TO achieved**	0.84	0.48–1.47	0.544
**ASA physical status ≥III**	1.85	1.15–2.96	0.011
**p/ypT ≥3 disease**	3.63	1.53–8.63	0.003
**Hemoglobin, per 1 g/dL**	1.03	0.91–1.18	0.599
**Neoadjuvant chemotherapy**	1.02	0.67–1.55	0.942
**C. Modified DUCA-TO Cox model**			
**Modified DUCA-TO achieved**	0.70	0.45–1.08	0.105
**ASA physical status ≥III**	1.73	1.08–2.78	0.023
**p/ypT ≥3 disease**	3.64	1.53–8.64	0.003
**Hemoglobin, per 1 g/dL**	1.04	0.92–1.17	0.534
**Neoadjuvant chemotherapy**	1.03	0.68–1.57	0.888

Values are hazard ratios with 95% confidence intervals from multivariable Cox proportional hazards models for overall survival, defined as time from surgery to death from any cause or last follow-up. Each model included the corresponding textbook outcome variable, ASA physical status ≥III, p/ypT ≥3 disease, preoperative hemoglobin, and neoadjuvant chemotherapy. The proportional hazards assumption was assessed using Schoenfeld residuals. Hazard ratios for textbook outcome achievement refer to achieved versus not achieved status; HR <1 indicates lower mortality risk. Original DUCA-TO used Clavien–Dindo grade ≥II morbidity as failure; Modified DUCA-TO used grade ≥III, with all other components unchanged. p/ypT refers to pathological T stage after upfront surgery or neoadjuvant chemotherapy, as applicable. HR, hazard ratio; CI, confidence interval; TOGS, Textbook Outcome in Gastric Surgery; DUCA-TO, Dutch Upper Gastrointestinal Cancer Audit textbook outcome; ASA, American Society of Anesthesiologists.

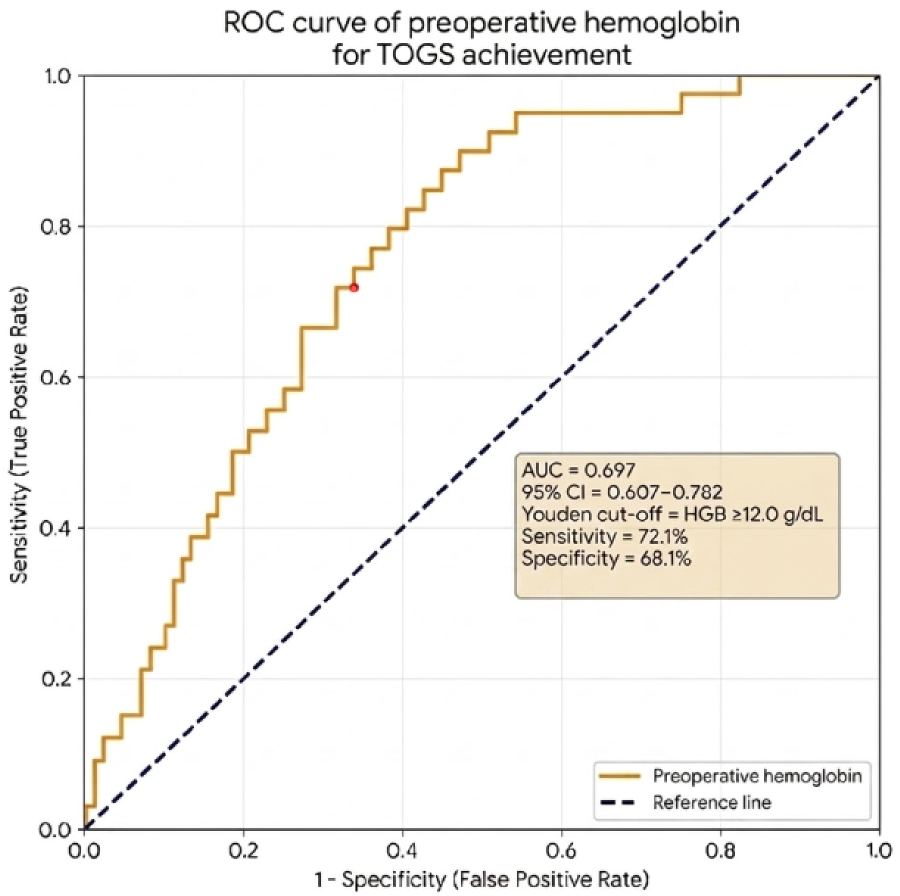

Supplementary Figure S1. Receiver operating characteristic curve of preoperative hemoglobin for predicting TOGS achievement. The ROC curve shows the discriminative performance of preoperative hemoglobin for TOGS achievement in the overall cohort. The area under the curve was 0.697 with a 95% confidence interval of 0.607–0.782. The optimal Youden cut-off was hemoglobin ≥12.0 g/dL, with 72.1% sensitivity and 68.1% specificity. The red dot indicates the Youden cut-off point. TOGS refers to the evaluated surgical/perioperative components of the GIRCG TOGS framework; the multimodality-treatment domain was not assessed. TOGS, Textbook Outcome in Gastric Surgery; ROC, receiver operating characteristic; AUC, area under the curve; CI, confidence interval.

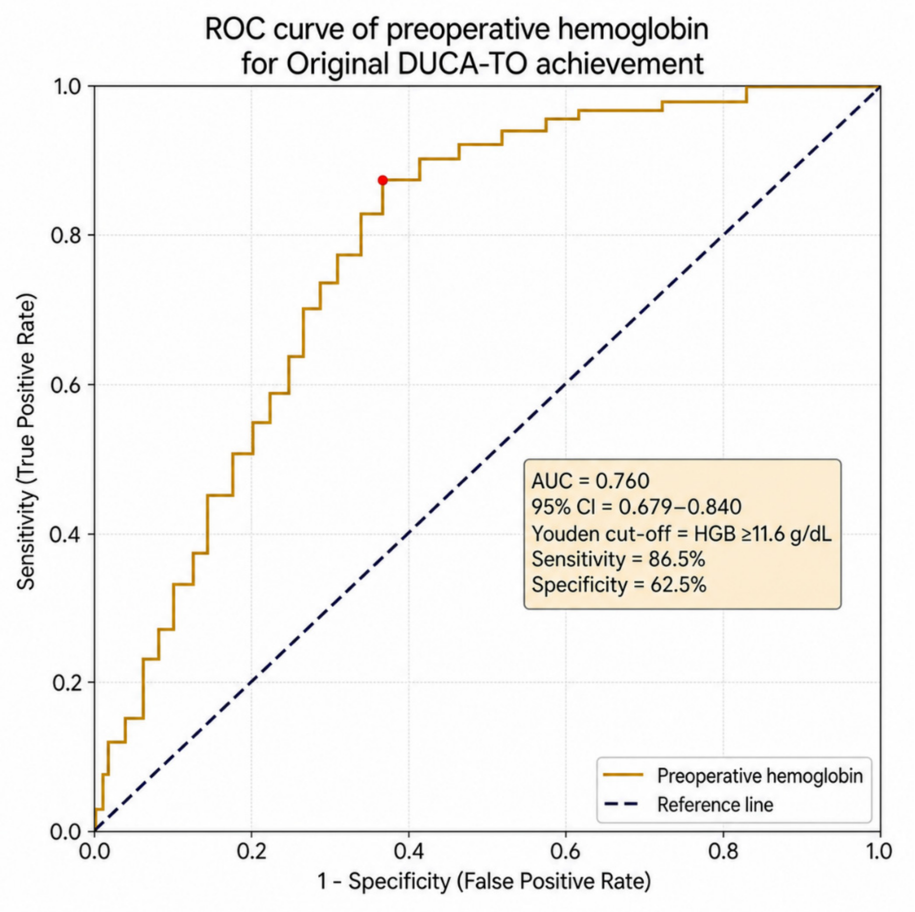

Supplementary Figure S2. Receiver operating characteristic curve of preoperative hemoglobin for predicting Original DUCA-TO achievement. The ROC curve shows the discriminative performance of preoperative hemoglobin for Original DUCA-TO achievement in the overall cohort. The area under the curve was 0.760 with a 95% confidence interval of 0.679–0.840. The optimal Youden cut-off was hemoglobin ≥11.6 g/dL, with 86.5% sensitivity and 62.5% specificity. Original DUCA-TO was calculated using Clavien–Dindo grade ≥II morbidity as failure. DUCA-TO, Dutch Upper Gastrointestinal Cancer Audit textbook outcome; ROC, receiver operating characteristic; AUC, area under the curve; CI, confidence interval.

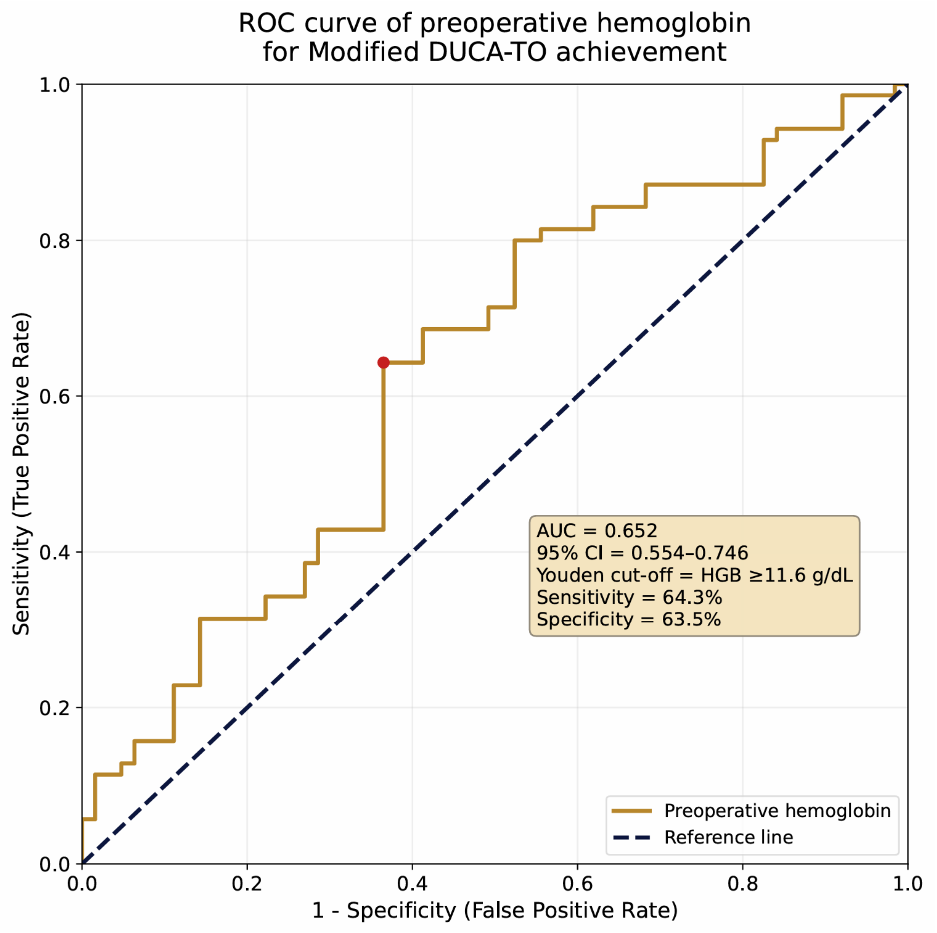

Supplementary Figure S3. Receiver operating characteristic curve of preoperative hemoglobin for predicting Modified DUCA-TO achievement. The receiver operating characteristic curve shows the discriminative performance of preoperative hemoglobin for Modified DUCA-TO achievement in the overall cohort. The area under the curve was 0.652, with a 95% confidence interval of 0.554–0.746. The optimal Youden cut-off was hemoglobin ≥11.6 g/dL, with 64.3% sensitivity and 63.5% specificity. Modified DUCA-TO was calculated using Clavien–Dindo grade ≥III morbidity as failure, with all other DUCA-TO components unchanged. DUCA-TO, Dutch Upper Gastrointestinal Cancer Audit textbook outcome; ROC, receiver operating characteristic; AUC, area under the curve; CI, confidence interval.
